# Mobile Health for Traumatic Brain Injury: A Systematic Review of the Literature and Mobile Application Market

**DOI:** 10.7759/cureus.5120

**Published:** 2019-07-10

**Authors:** Edward Christopher, Kareem W Alsaffarini, Aimun A Jamjoom

**Affiliations:** 1 Neurological Surgery, College of Medicine and Veterinary Medicine, University of Edinburgh, Edinburgh, GBR; 2 Neurological Surgery, College of Life Sciences and Medicine, University of Aberdeen, Aberdeen, GBR; 3 Neurological Surgery, Centre for Clinical Brain Sciences, University of Edinburgh, Edinburgh, GBR

**Keywords:** concussion, mhealth, mobile technology in healthcare, traumatic brain injury (tbi)

## Abstract

Traumatic Brain Injury (TBI) is a growing public health issue with an increasing burden of disease globally. TBI can lead to significant motor, cognitive and emotional deficits. Mobile health (mHealth) is a promising technology to help diagnose and manage patients with TBI. The aim of this study was to systematically examine and classify available TBI mobile applications (apps) and critically appraise the literature underpinning mHealth for the management of TBI. Two major app markets (Apple and Google Play) were systematically searched. Included apps were classified and had data extracted. Coupled to this, a systematic search of the literature (MEDLINE, Web of Science, Scopus, PsycINFO) was performed examining the effectiveness of mHealth interventions in helping patients manage their symptoms after TBI (registered with PROSPERO: CRD42018107386). From 1296 apps, 53 met our inclusion criteria. The top three functions were TBI screening, education and biomechanics monitoring. Twenty-six apps (49.1%) focused on sports-related concussion. Eight apps (15.1%) were gamified and 12 apps (22.6%) connected to an external device. From the literature, a total of eight articles were included of which four (50%) were case series, two (25%) were feasibility/pilot studies, one (12.5%) was a case report, and one (12.5%) was a randomised controlled trial. The median number of patients was seven (1 - 43). There is a small number of mobile apps for TBI, mostly focusing on sports-related concussion. At present, the uptake and application of these apps as a management aid is limited and the evidence for their usefulness in TBI remains low.

## Introduction and background

Traumatic Brain Injury (TBI) is a growing public health issue with an increasing burden of disease globally [1]. There is a spectrum of TBI severity with severe cases leading to significant motor, cognitive, and emotional deficits. Even mild injuries, which constitute over 80% of cases, can lead to post-concussional symptoms (this includes a range of symptoms that come under four categories: somatic, cognitive, emotional and sleep related) which can impair patients’ function [2]. The challenges in TBI management are numerous including promoting awareness and public education. For concussion or mild TBI, there is a big need for early identification of patients and providing support for post-concussional symptoms. While for more severe TBIs there is a need to deliver effective motor and cognitive rehabilitation. Mobile technology provides a potential means to meet a number of these challenges in TBI care. Over recent years, mobile health (mHealth) has emerged as an increasingly promising area for supporting patients and healthcare professionals in a number of diseases such as diabetes and mental health problems [3,4]. mHealth refers to the practice of medicine by a mobile device such as a mobile phone, tablet computer and, more recently, wearable sensors. The mobile technology has been used in screening for sports-related concussion and in delivering TBI education [5]. However, despite the promise of mHealth in TBI, there has not been a comprehensive analysis of available mobile applications (apps) and a review of the literature. In this study, we aimed to examine the available commercial mobile apps for TBI and examine the evidence underpinning their use for managing TBI symptoms. To do this, we systematically examined the two largest mobile app markets for apps focused on TBI. Coupled to this, we conducted a systematic review of the literature to understand the underlying evidence for mHealth interventions for helping patients manage their symptoms after a TBI.

## Review

Materials and Methods

A systematic review of the mobile app market

A systematic review for available mobile apps was performed by searching the mobile app markets Google Play and the Apple App Store between June to December 2017. The mobile app markets were each systematically searched using the following keywords: ‘concussion’, ‘head injury’, ‘traumatic brain injury’ or ‘brain injury’. Initial screening was based on the app name and description provided by the developers. Apps were reviewed independently for eligibility by two investigators (EC - Edward Christopher and AJ - Aimun Jamjoom). Apps were eligible for inclusion if they had one of the following functions: TBI education (defined as the provision of written or graphical information about TBI, its diagnosis and its management); TBI symptom management and/or tracking (defined as the functionality permitting users to input data about their symptoms using the app); TBI assessment/screening tool (defined as the functionality permitting users to input data that stratifies/detects/diagnoses TBI or concussion using the app) and TBI biomechanics measurement (defined as the functionality permitting the app to detect, measure and record biomechanical forces related to TBI). Apps that were not in English were excluded from further analyses. Apps that met our inclusion criteria were installed and reviewed independently by two investigators (EC and AJ). We extracted a number of data points including: name, availability in Android or iOS devices or both, main function, if the app is sports-focused, connectivity to external device, gamification, user rating, price in pound sterling (£), number of downloads (until February 2018) and Pan European Game Information rating (PEGI) which is an age recommendation (PEGI3; PEGI7; PEGI12; PEGI16 and PEGI18). If cross-platform duplicates were found, only the most recently updated version of the app was included for analyses.

A systematic review of the literature

We conducted a systematic review with the aim of answering the following question: are mHealth interventions effective in managing patient symptoms after a TBI? The systematic review was registered on the PROSPERO database (CRD42018107386 - http://www.crd.york.ac.uk/PROSPERO/display_record.php?ID=CRD42018107386) and we used the PRISMA guidelines for reporting of this systematic review [6]. A literature search was performed using MEDLINE (accessed by PubMed), Web of Science, Scopus and PsycINFO in August 2018. A search strategy combining a number of TBI-related phrases was used: (“Traumatic brain injury”, "Concussion", "TBI" + “mHealth", "Mobile phone", "Smartphone", "iPhone", "Mobile app") (Table 1). We aimed to include clinical studies that looked at the impact of mHealth in managing patient symptoms after a TBI. We defined mHealth as any mobile technology including smartphones or handheld devices, mobile applications, mobile phone text messaging interventions, phone calendar/reminders and sensors linked to mobile devices. Databases were searched from inception to August 2018. The study had to include clinical outcome measures related to TBI symptomology (somatic, emotional, cognitive and sleep). The studies had to focus on TBI patients exclusively and we excluded studies looking at mixed populations of patients with acute brain injuries (such as stroke or post-traumatic stress disorder). We included only English language articles and excluded preclinical studies. Our inclusion criteria of study design included randomised controlled trials (RCT), observational studies (case reports, case series, cohort and case-control studies) and pilot/feasibility studies. For RCTs, we examined the degree of bias using the risk of bias tool found in the Cochrane handbook for systematic reviews of interventions [7]. For case reports or case series, we used a recently proposed framework by Murad and colleagues to examine methodological quality based on four criteria: selection, ascertainment, causality and reporting [8]. Initial screening was based on titles and abstracts which were reviewed by two independent investigators (KA - Kareem Alsaffarini and EC). Subsequently, the two investigators independently evaluated full-text articles and determined eligibility. Any discrepancies were examined by a third investigator (AJ) who made the final decision on the application of inclusion criteria. Included studies had the following data extracted and collated in a data-sheet: author, publication year, type of mHealth Intervention, TBI severity (mild/moderate/severe), number of patients, duration of follow up, outcome measures and key findings. The reference lists of included studies were also reviewed, and any additional eligible studies were included in the review.

**Table 1:** Search strategy for systematic review Search terms (1-8) were added in pairs and their results combined in search strategy (9-11) TBI: traumatic brain injury; Mobile app: mobile application

Code	Search term
1	“Traumatic Brain Injury”
2	“Concussion”
3	“TBI”
4	“Mhealth”
5	"Mobile Phone"
6	"Smartphone"
7	"iPhone"
8	"Mobile App"
9	(1 AND 4) + (1 AND 5) + (1 AND 6) + (1 AND 7) + (1 AND 8)
10	(2 AND 4) + (2 AND 5) + (2 AND 6) + (2 AND 7) + (2 AND 8)
11	(3 AND 4) + (3 AND 5) + (3 AND 6) + (3 AND 7) + (3 AND 8)

Results

A systematic review of the mobile app market

**Figure 1:**
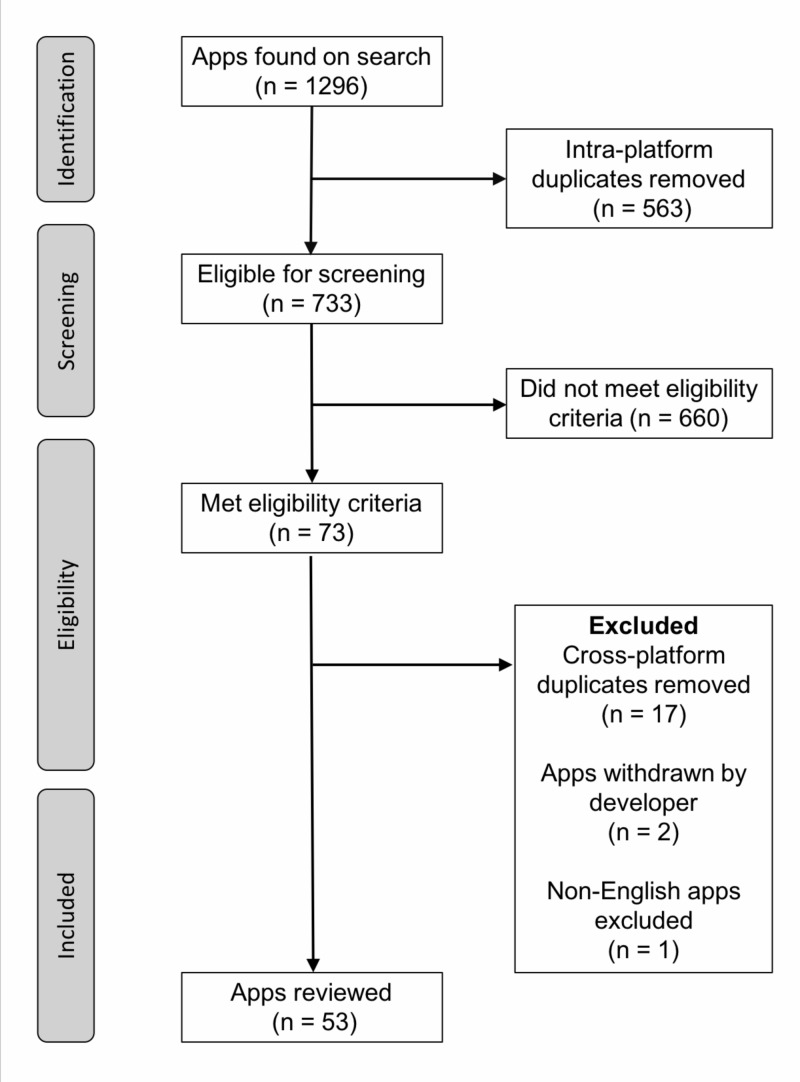
Flow chart of included mobile apps in systematic review of mobile app market Apps: mobile applications

Our search strategy returned 1296 apps of which 563 apps (43.4%) were intra-platform duplicates. We removed these duplicates and of the remaining 733 apps, 73 apps (10.0%) met our inclusion criteria. We removed 17 cross-platform duplicates and excluded three apps: two were withdrawn from the platform by the developers during the course of the study and another for being non-English. The final inclusion list consisted of 53 apps (Figure 1). The 53 apps reviewed in this study are outlined in Table 2. Of the apps, 29 (54.7%) were exclusively available on Google Play, 10 apps (18.9%) were only available on App Store and 14 apps (26.4%) were available on both app markets. The majority of apps were updated in the last two years: 2017 (45.3%) followed by 2016 (26.4%). We classified apps according to their main function related to TBI. The most common function in 18 of the apps (34.0%) was as a TBI assessment/screening tool. The three top app functions were: delivery of TBI education (28.3%), detection of TBI biomechanics forces (20.8%) and symptom management and/or tracking (17.0%). Eight apps (15.1%) were gamified and 12 apps (22.6%) connected to an external device.

**Table 2:** Summary of included mobile app characteristics App: mobile application; TBI: traumatic brain injury; CDC: Centers for Disease Control; CNS: central nervous system

Name	Operating system	External device	Gamification	Downloads	Main function	Price (£)	Sports-focused
Accident Concussion Scale (ACS)	iOS	-	-	-	TBI assessment/ screening tool	0	-
AI Sport	Android	+	-	10-50	TBI biomechanic sensor	0	+
AoS Concussion Sensor Main App	Android	+	-	1-5	TBI biomechanic sensor	0	+
Barrow Brainball	Android	-	+	1000-5000	TBI education	0	+
BICS	Android	-	-	1-5	TBI assessment/ screening tool	4.92	-
Brain Injury	Android, iOS	-	-	100-500	Symptom management ± tracking	0	-
CDC Heads Up Concussion Safety	Android	-	-	1000-5000	TBI education	0	-
CDC Heads Up Rocket Blades – The Brain Safety Game	Android, iOS	-	+	-	TBI education	0	-
CNS Mobile	Android, iOS	-	-	5000-10000	TBI education	0	-
Concussion Assessment & Response	Android, iOS	-	-	500-1000	TBI assessment/ screening tool	3.10	+
Concussion Awareness	iOS	-	+	-	TBI education	0	+
Concussion Buddy	iOS	-	-	-	Symptom management ± tracking	0	-
Concussion Coach	Android	-	-	500-1000	Symptom management ± tracking	0	-
Concussion Diagnostic Tool	iOS	-	-	-	TBI assessment/ screening tool	0	+
Concussion Ed	iOS	-	-	-	Symptom management ± tracking	0	-
Concussion Injuries	Android	-	-	50-100	TBI education	0	-
Concussion Management	Android, iOS	-	-	1000-5000	TBI education	0	-
Concussion Recognition & Response	Android, iOS	-	-	10000-50000	TBI assessment/ screening tool	0	+
Concussion Signs and Symptoms	Android	-	-	1000-5000	TBI education	0	-
Concussion Test & Tracker by SportGait	Android	-	-	100-500	Symptom management ± tracking	0	+
CSx HeadGuard	Android, iOS	-	-	100-500	TBI assessment/ screening tool	0	+
Don’t Pull The Plug!	Android	-	+	50-100	TBI assessment/ screening tool	0	-
FACT Concussion Test	Android	-	-	100-500	TBI assessment/ screening tool	0	+
FirstResponder^TM^ Concussion App	Android	-	-	500-1000	TBI assessment/ screening tool	0	+
Head Case	Android	+	-	50-100	TBI biomechanic sensor	0	+
Head Injury Association	iOS	-	-	-	TBI education	0	-
Headcheck	iOS	-	-	-	TBI assessment/ screening tool	0	-
HeadTek	Android	+	-	100-500	TBI biomechanic sensor	0	+
HEADWays	Android	-	-	100-500	TBI education	0	-
HHN Impact Monitor	iOS	+	-	-	TBI biomechanic sensor	0	+
HitCheck: Sideline Concussion Testing	Android, iOS	-	+	1000-5000	TBI assessment/ screening tool	0	+
i1 Vector	Android	+	-	10-50	TBI biomechanic sensor	0	+
ICEdot	Android	+	-	1000-5000	TBI biomechanic sensor	0	+
ImPACT Passport	Android, iOS	-	-	5000-10000	Symptom management ± tracking	0	-
InVinci-BULL Shockbox	Android	+	-	1-5	TBI biomechanic sensor	3.13	+
King-Devick Test with Mayo Clinic	iOS	-	-	-	TBI assessment/ screening tool	0	+
MACE Concussion Evaluation	Android	-	-	50-100	TBI assessment/ screening tool	0	-
Max Impact	Android, iOS	-	+	10-50	Symptom management ± tracking	0	-
Medrills: Skull and Brain	Android, iOS	-	-	10-50	TBI education	3.13	-
mTBI Pocket Guide	Android	-	-	10000-50000	TBI education	0	-
PlayerMD Biometric Platform	Android	+	-	100-500	TBI biomechanic sensor	0	+
Pocket TBI	Android	-	-	5000-1000	TBI education	0	-
Rancho Los Amigos Scale	Android	-	-	5000-10000	TBI assessment/ screening tool	0	-
Rebound: Beating Concussions	Android, iOS	-	+	10-50	TBI education	0	+
Return2Play for Concussion	Android	-	-	500-1000	Symptom management ± tracking	0	+
SACTool Beta	Android	-	-	500-1000	TBI assessment/ screening tool	0	-
Shockbox	Android	+	-	1000-5000	TBI biomechanic sensor	0	+
SwiftReact	Android	+	+	10-50	Symptom management ± tracking	0	-
TBI Prognosis Calculator	Android, iOS	-	-	1000-5000	TBI assessment/ screening tool	0	-
The Blast Gauge System App	Android	+	-	10-50	TBI biomechanic sensor	0	-
World Rugby Concussion	Android, iOS	-	-	1000-5000	TBI education	0	+
X2 ICE	iOS	-	-	-	TBI assessment/ screening tool	0	+
XLNTbrain-mobile	Android	-	-	500-1000	TBI assessment/ screening tool	0	+

In terms of the target audience, nine apps (17.0%) were designated for healthcare professionals, 18 apps (34.0%) were designated for patients and 26 apps (49.1%) were designated for athletes. The majority of apps (66.0%) were rated PEGI3, followed by PEGI7 (13.2%), PEGI12 (5.7%), and PEGI18 (1.9%). Seven apps (13.2%) were unrated. In terms of user rating, the median rating was 4.2 stars out of five [Interquartile range (IQR) 3.5 - 5, n=35] with the median number of ratings per app being five (IQR 2 - 13, n=35). We excluded 18 apps from these analyses owing to them having no user rating at all. Price ranged from free to £4.92 but most apps (92.5%) were free. Google Play provides the number of downloads for each app in ranges. Taking the middle of the range as the number of downloads for each app, the median number of downloads was 300 (IQR 75 - 3000, n=43) for apps available on Google Play. The Apple App Store does not provide download metrics and thus we were unable to analyse these data for apps exclusively available on App Store (n=10).

Sports-focused apps

We examined in detail the 26 apps that were designated for athletes since they formed the majority in our final inclusion list. Fifteen apps (57.7%) were exclusively available on Google Play, five apps (19.2%) were exclusively available on App Store and six apps (23.1%) were available on both. We classified these sports-focused apps according to their main function: 10 apps (38.5%) focused on TBI assessment/screening tool, four apps (15.4%) delivered sports-related TBI education and two apps (7.7%) focused on symptom management ± tracking. Four apps (15.4%) were gamified. Ten apps (38.5%) could accommodate connectivity to an external device. Of these, six apps could accommodate only one device, three apps could accommodate two devices, and one app could accommodate up to four external devices. External devices included mouth guards, impact sensors, headbands, accelerometers, gyroscopes, thermometers, helmets, and skullcaps. In terms of user rating, the median rating was 4.2 stars out of five (IQR 3.9 - 5, n=17) with the median number of ratings per app being five (IQR 2 - 13, n=17); we excluded nine apps from these analyses owing to them having no user rating. Price ranged from free to £3.13 but most sports-focused apps (92.3%) were free. Google Play provides the number of downloads for each app in ranges. Taking the middle of the range as the number of downloads for each app, the median number of downloads was 300 (IQR 75 - 3000, n=21) for apps available on Google Play. App Store does not provide download metrics and thus we were unable to analyse these data for apps exclusively available on App Store (n=5).

A systematic review of the literature

**Figure 2:**
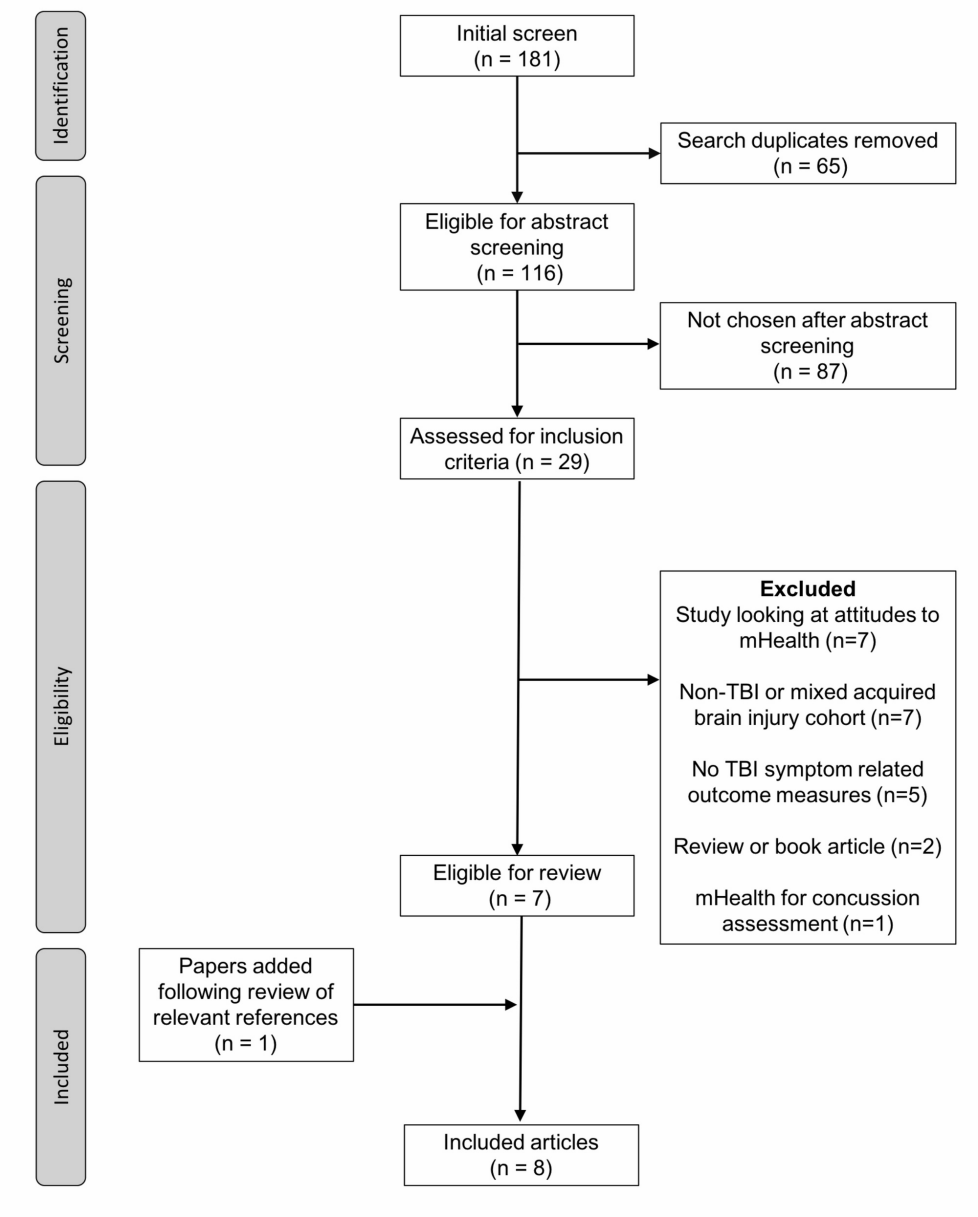
Flow chart of included studies in systematic review of literature TBI: traumatic brain injury

A total of 181 articles returned using our search strategy in August 2018. After removing duplicates, a total of 116 papers remained. The abstract screening was performed, and 29 papers were chosen for a more detailed review of the full article. Seven articles met our final inclusion criteria from which one further article was added after an examination of the reference lists (Figure 2). A summary of the eight included studies can be found in Table 3 [8-15]. Of the included studies, four (50%) were case series, two (25%) were feasibility/pilot studies, one (12.5%) was a case report, and one (12.5%) was an RCT [14]. We examined the risk of bias in the RCT using the Cochrane Risk of Bias Tool: random sequence generation (low risk), allocation concealment (high risk), blinding of participants and personnel (high risk), blinding of outcome assessment (high risk), incomplete outcome data (low risk) and selective reporting (low risk). Based on the Murad criteria, we found that one of the case reports was high quality while the rest were of medium quality. The median number of patients was seven (1 - 43). The most common investigation in five (62.5%) articles was the role of mHealth intervention as a memory aid (including goal setting) after TBI. The remaining three (37.5%) studies looked at mHealth intervention as a tool for symptom/mood management after TBI. Seven studies (87.5%) examined a mobile app as the mHealth intervention in TBI.

**Table 3:** Summary of included mHealth in TBI studies mHealth: mobile health; EMA: ecological momentary assessment; SPAN: Social Participation and Navigation; PHQ-4: patient health questionnaire 4; EMQ: Everyday Memory Questionnaire; TUQ: Telehealth Usability Questionnaire; YSR: Youth Self Report; CBCL: Child Behavior Checklist; RPQ: Rivermead Post-concussional Questionnaire; PTSD: Post-traumatic stress disorder; LOT-R: Life Orientation Test– Revised; CES-DC: Center for Epidemiological Studies–Depression Child

Author	mHealth Intervention	Study Type	TBI Severity	Number of Patients	Outcome Measures	Duration of follow-up	Quality	Key Findings
Baldwin [9]	Google Calendar on mobile phone	Case report	Severe	One	% target events forgotten; The Beliefs about Memory Aids Questionnaire; Revised Everyday Memory Questionnaire (EMQ)	Six weeks of baseline data and six weeks of intervention data	High	Objective improvement in target events following intervention; Increased personal and treatment beliefs post-intervention; Inappropriate beliefs about memory aids decreased
Bos [10]	Mobile apps (Google calendar, Gtasks, SimpleCalendar and Calendar Snooze)	Case Series	Moderate to Severe	Nine	Test of Memory Malingering; The Rivermead Behavioural Memory Test–II; Message time task: place call at the scheduled time; Message content task: address question in a message; Postcard task: send postcard at allocated time; Depression Anxiety and Stress Scales 21 item version; Comprehensive Assessment of Prospective Memory; Community Integration Questionnaire	Eight weeks	Medium	Participants using a smartphone showed improvements in their ability to complete assigned memory tasks accurately and within the assigned time periods; 86% showed improvements in their ability to perform assigned functional memory tasks using the smartphone; Variable improvement in postcard task between participants; Over 90% performance on both message time and message content was reached by three participants with smartphone
Cruz [11]	Mobile application (Google calendar)	Case series	Mild and Severe	Two	Canadian Occupational Performance Measure; Patient Competency Rating Scale; The Hospital Anxiety and Depression Scale; Wechsler Adult Intelligence Scale; Rey Complex Figure Test; Cambridge Prospective Memory Test; Rivermead Behavioural Memory Test – Third Edition; Modified Six Elements Test from the Behavioural Assessment of the Dysexecutive Syndrome	Approximately three months for each participant	Medium	Mobile text reminders led to an improvement in performance and satisfaction; Reminders had to be tailored to the individual to maximise impact; The use of different reminder modalities helps improve the effectiveness
Juengst [12]	Tracking mood-related symptoms using a smartphone application (iPerform platform)	Feasibility Study	Mild to Severe	20	Compliance measured the total number of completed assessments divided the total number of scheduled assessments; Satisfaction measured with 7-point Likert scale assessing usability and satisfaction with the application; Telehealth Usability Questionnaire (TUQ)	Eight weeks	Medium	Participants correctly completed 73.4% of all scheduled assessments; Participants reported high satisfaction with smartphone applications and found them easy to use; Comparison of assessments obtained via telephone-based interview and EMA demonstrated high correlations; Participants reported high satisfaction across the eight weeks of using the iPerform application
Narad [13]	App-based coaching intervention (SPAN) for adolescents with TBI	Case Series	Mild to Severe	Four	Patient/parent satisfaction using a brief questionnaire to assess ease of use and helpfulness of various aspects of the program using a Likert-type scale from one to five; Number and type of goals achieved; Participants completed the Youth Self Report (YSR) and their parents completed the Child Behavior Checklist (CBCL)	Four weeks	Medium	All participants felt app provided appropriate scaffolding for goal setting and management; Two of the four participants completed all steps, achieved their goals and identified additional goals to work after completion of the SPAN program; No statistically significant differences between pre- and post-intervention scores for social competency and social problems
Stapleton [14]	Using a ‘reminders’ function on a mobile phone as a compensatory memory aid	Case Series	No data	Five	The Speed and Capacity of Language Processing; The Spot the Word Test; The Rivermead Behavioural Memory Test; The map search from the Test of Everyday Attention; The Tower Test; Individuals and/or their carers recorded their memory successes in achieving the target behaviours throughout the course of the study	Randomization of different lengths of baseline data (2, 3 and 4 weeks) seven weeks of intervention data followed by two weeks of baseline data and another two weeks of intervention data	Medium	For two participants, there was an increase in the target behaviours achieved when the phone was used; The percentage of target behaviours achieved did not return to initial baseline levels when the phone was removed; The participants who did not benefit from the mobile phone differed as they fell in the category of ‘severe memory impairment’, were significantly impaired on an assessment of executive functioning and required 24-hour care.
Suffoletto [15]	Timed SMS symptom assessments with self-care support messages in individuals with TBI	Randomised Control Trial	Mild	43 (18 intervention; 25 control)	Rivermead Post-concussional Questionnaire (RPQ); Primary care post-traumatic stress disorder (PTSD) screen; Anxiety and depression were measured using the PHQ-4; Painkiller use; Participants in the Intervention groups were asked to complete additional survey questions to measure their perception of the SMS program	14 days	Random sequence generation (low risk), allocation concealment (high risk), blinding of participants and personnel (high risk), blinding of outcome assessment (high risk), incomplete outcome data (low risk) and selective reporting (low risk)	Compared with the control group, intervention participants trended to lower odds of reporting headache, concentration difficulty, and irritability or anxiety; There were no significant differences in RPQ score, proportion meeting post-concussion disease criteria, PTSD, anxiety or depression between intervention and control groups; All of the intervention participants thought that the messages were at least somewhat useful to help them understand their symptoms, and 93% found them at least somewhat useful to help them self-manage their symptoms.
Worthen- Chaudhari [16]	A mobile health application that employs elements of social game design as a complement to medical care for unresolved concussion symptoms	Phase I (feasibility study) Phase II (non-randomized, open label, controlled study)	Mild	Phase I: 20; Phase II: 22 (12 intervention; 10 control)	Phase I: Number of participants completing the intervention relative to all enrolled; App use (% Play), expressed as percent of target dose in the first three weeks of intervention; Satisfaction with intervention, rated on a seven-point Likert scale; Barriers to compliance Phase II: SCAT-3 symptom checklist score to assess concussion symptom severity; Optimism, as measured by the Life Orientation Test– Revised (LOT-R); Depression, as measured by the Center for Epidemiological Studies–Depression Child (CES-DC)	Not specified	Medium	A majority of participants completed the intervention (14 of 20) with high use and satisfaction; Significant improvement in symptoms and optimism for the experimental cohort compared to control; There was no significant difference between control and experimental cohort for depression scores

Discussion

mHealth is increasingly used in medical practice and has the potential to improve healthcare delivery through enhanced communication, data capture, patient monitoring, education and the delivery of digital interventions at scale [17]. A systematic review looking at the role in mHealth for rehabilitation identified seven studies that focused on the use of mHealth for TBI rehabilitation [18]. The study showed that mHealth interventions have been investigated for TBI rehabilitation in a range of areas including reminder support, managing mood-related symptoms and cognitive rehabilitation. TBI is a major cause of injury-related death and disability globally [1]. However, the spectrum of the disease from mild to severe means different patient cohorts require different support strategies. All TBI patients require support with understanding and managing their symptoms such as headaches, mood disturbance and difficulty sleeping. However, patients with more severe injuries with cognitive and motor deficits require tools to support them such as reminder notifications, cognitive and motor rehabilitation. 

Mobile devices are becoming increasingly accessible and they have the potential to play a central part in TBI education, screening, and management. Our systematic review demonstrated that a relatively small number of TBI apps are currently commercially available. This is considerably smaller in comparison to other pathologies such as diabetes and depression which were found to have over 1000 apps [19]. This difference is disproportionately large considering the global burden of TBI and particularly as a recent Lancet Neurology Commission highlighted the suboptimal state of TBI diagnosis, monitoring, and management in parts of the world; areas where mHealth could provide solutions [20].

Of the included mobile apps, we found a low level of user engagement with a median of 300 downloads and only an upper quartile of 3000. Given the epidemiology of TBI and its growing public profile, this shows very limited uptake of these mobile apps among patients and healthcare professionals. Wong and colleagues looked at the attitudes of patients with TBI towards the use of a mobile phone and found positive views regarding the benefits of mobile phones as a means of communication and memory aid [21]. It does suggest however that the current commercial TBI app market landscape is not being actively used by the target audience. This may be because TBI patients’ needs are being met by other more established programmes such as reminder/calendar apps native to the patients’ mobile phones. Coupled to this, medical apps are not tightly regulated and users may question their accuracy. This was highlighted by a recent systematic review which looked at the compliance of concussion assessment apps and found a wide range of compliance rates with a concussion assessment tool [5]. This is concerning, given that the most common app function in our review was TBI screening/assessment. There are growing efforts to provide organised peer review of apps in platforms such as TopOrthoApps and iMedicalApps. The National Health Service (NHS) is currently running the beta site Digital Apps Library which lists apps which meet the NHS quality standards for clinical effectiveness, safety, usability and accessibility and has evidence to support its use.

In the second stage of this study, we performed a systematic review of the literature to look for the evidence underpinning the use of mHealth in managing patient symptoms after TBI. We found only eight studies of which five were either case reports or case series reflecting a very limited evidence base. These studies provide anecdotal evidence of the benefits of mobile apps for patients with TBI. A number of these reports used baseline control data and compared it to mHealth intervention phases which showed varying degrees of improvement in task achievement and satisfaction. Of the controlled studies, Worthen-Chaudhari et al. looked at a gamified mobile app as a tool to help improve post-concussional symptoms in adolescents [15]. In this feasibility study, the investigators found the majority of participants completed the intervention and that there was an improvement in symptom burden in the intervention group. This contrasted to the only RCT in the included studies which looked at a short messaging service (SMS) based intervention for patients following concussion [15]. The investigators found that an intervention of text message-based advice on symptom control did not lead to a significant reduction in symptom burden. Collectively, this review has shown a wide range of mHealth interventions have been tested for patients with TBI. The anecdotal evidence shows potential for mHealth interventions to help individuals with TBI. However, the evidence base is limited, and we conclude that there is currently insufficient data to support the use of mHealth in managing symptoms after TBI.

Our study has a number of limitations. We only looked at iOS and Android markets which may mean we missed some mobile apps for other platforms such as Microsoft or Blackberry. Also, we only examined studies focusing on managing TBI patients and excluded other studies that look at different aspects of mHealth in TBI care. Despite these drawbacks, we believe that the data presented in this study is the most comprehensive examination of mHealth in TBI to date. It demonstrates a limited number of commercially available mobile apps that have little uptake from patients and healthcare professionals based on the download numbers. Coupled to this, we found that the evidence base underpinning mHealth interventions in TBI to be mainly anecdotal in the form of case reports and case series. 

## Conclusions

Despite a growth in the use of mHealth for other diseases, our study found only a small number of mobile apps for TBI with limited uptake based on download metrics. The majority of these focused on sports-related concussion and were aimed at either concussion screening or TBI education. Coupled to this, the evidence underpinning mHealth in TBI is limited with a dearth of large clinical trials examining this area. Given the range of care needs TBI patients have, there is scope to develop TBI-specific mHealth interventions that aim to help patients that are robustly tested for clinical effectiveness. 
